# Stressful life events and serum triglyceride levels: the Cardiovascular and Metabolic Diseases Etiology Research Center cohort in Korea

**DOI:** 10.4178/epih.e2021042

**Published:** 2021-06-09

**Authors:** Naharin Sultana Anni, Sun Jae Jung, Jee-Seon Shim, Yong Woo Jeon, Ga Bin Lee, Hyeon Chang Kim

**Affiliations:** 1 Department of Public Health, Graduate School, Yonsei University, Seoul, Korea; 2 Department of Preventive Medicine, Yonsei University College of Medicine, Seoul, Korea; 3 Cardiovascular and Metabolic Diseases Etiology Research Center, Yonsei University College of Medicine, Seoul, Korea

**Keywords:** Psychological stress, Lipids, Hypertriglyceridemia

## Abstract

**OBJECTIVES:**

Elevated serum triglyceride levels are a risk factor for developing cardiovascular disease. A number of studies have demonstrated a positive association between psychological stress and serum triglyceride levels. However, there is limited evidence regarding the impact of stressful life events (SLEs) on serum triglyceride levels in the healthy population. Therefore, we evaluated the independent association between SLEs and serum triglyceride levels in a middle-aged Korean population.

**METHODS:**

We analyzed a sample of 2,963 people (aged 30-64 years; 36% men) using baseline data from the Cardiovascular and Metabolic Diseases Etiology Research Center (CMERC) cohort study. The Korean version of the Life Experience Survey questionnaire was used to measure the presence and positive/negative impact of SLEs. Hypertriglyceridemia was defined as a fasting serum triglyceride level of ≥ 150 mg/dL.

**RESULTS:**

Of the 2,963 participants, 33.1% reported at least 1 SLE over the past 6 months and 24.8% had hypertriglyceridemia. Even after adjusting for potential confounders, the serum triglyceride level was significantly associated with the total number of SLEs in men (3.333 mg/dL per event; p= 0.001), but not in women (0.451 mg/dL per event, p= 0.338). Hypertriglyceridemia was also associated with having 4 or more SLEs with positive effects (odds ratio [OR], 2.57; 95% CI, 1.02 to 6.46) and 4 or more SLEs with negative effects (OR, 1.99; 95% CI, 1.16 to 3.41) in men.

**CONCLUSIONS:**

Our findings suggest that SLEs may increase the risk of hypertriglyceridemia in middle-aged men.

## INTRODUCTION

Dyslipidemia, which consists of increased blood total cholesterol, low-density lipoprotein (LDL)-cholesterol, and triglyceride levels and decreased high-density lipoprotein (HDL)-cholesterol levels, is one of the most strongly contributory risk factors for cardiovascular disease. High triglyceride levels are now considered an independent risk factor of cardiovascular disease [1]. Several studies have reported that a high serum triglyceride level alone or in combination with a low HDL-cholesterol or high LDL-cholesterol level is associated with atherosclerotic cardiovascular disease [2,3].

There is increasing evidence suggesting that physical and psychological stress can affect human physiological parameters, including the lipid profile [4], and increase the risk of cardiovascular disease and mortality [5]. It is hypothesized that the activity of the hypothalamic-pituitary-adrenal (HPA) axis can be increased due to chronic stress, and the resultant increase in cortisol levels leads to an upsurge in both insulin resistance and abdominal obesity with lipids and blood pressure, thereby facilitating the development of metabolic syndrome [6]. Additionally, physical stress generated through physical activity can also affect the lipid profile [7]. The workplace is an important source of both mental stress and physical stress. Several studies have been conducted among specific types of workers to evaluate the effect of stress or stressful events in the workplace on metabolic abnormalities [8,9]. Although the findings of those studies strongly support the adverse metabolic effects of job-related stress, those findings cannot be generalized to stress in other situations. There is still scarce evidence for independent associations between psychological stress from life events and the lipid profile.

The Korean population is known to have a high level of psychological stress and an exceptionally high rate of suicide. A recent Korean cohort study, involving 1,071 participants aged 15-16 years who were followed up for an average of 6 years, reported that consistently low or increased total cholesterol levels during adolescence predicted an elevated risk of depressive symptoms in early adulthood [10]. However, the effects of life-related stress on the blood lipid profile have not been investigated previously in the healthy Korean population. Therefore, we evaluated the association between stressful life events (SLEs) and serum triglyceride levels, as a component of the lipid profile, in a middle-aged Korean population.

## MATERIALS AND METHODS

### Study population

The Cardiovascular and Metabolic Diseases Etiology Research Center (CMERC) cohort data were used for this study. The recruitment and baseline examinations of the CMERC community cohort were performed from 2013 to 2018 at 2 study centers: center 1 was located in Seoul, and center 2 was located in Suwon, Gyeonggi Province [11]. Participants were recruited mostly through advertisements in regional newspapers, promotional posters in public areas, or through the acquaintances of study participants. Data from center 1 were selected for the present study because of accessibility. The participants from center 1 were residents of Seoul and its western suburbs, tended to be more educated, were less likely to be married, and had higher blood lipid levels than those from center 2. Nevertheless, there were no significant differences in SLEs between the 2 centers (Supplementary Material 1). A total of 4,060 men and women aged 30-64 years without a history of myocardial infarction, heart failure, stroke, or cancer completed examinations at center 1. After excluding those who had missing data for the study variables (n= 495), lipid-modifying drug users (n= 461), and patients diagnosed with diabetes (n= 216) or kidney disease (n= 7), 2,963 people were left, yielding the sample of the present study (Supplementary Material 2).

### Measurements

In the CMERC cohort, trained interviewers examined subjects using the Life Experiences Survey Questionnaire [12], which has been translated to Korean and validated for the Korean population [13]. Participants were asked about a total of 50 SLEs, including marital status, death of a close family member, sexual difficulties, or conflict with colleagues, that occurred any time during the last 6 months of the participants’ life. The distribution of 50 types of SLEs by frequency (yes or no) and their impact (positive impact, no impact, and negative impact) are provided in Supplementary Material 3. The participants were instructed to mark whether they experienced those life event items and were given a 7-point scale ranging from +3 (extremely positive effect) to -3 (extremely negative effect). In our study, we calculated the total number of SLEs, the number of SLEs with positive effects, and the number of SLEs with negative effects, and then divided the numbers into 4 groups (no, 1, 2-3, and ≥ 4 events). Furthermore, we considered the SLE scores as a measure of the direction and strength of each event. We calculated the sums of positive or negative effect scores and divided them into 4 groups similar to those calculated for events (scores of 0, 1, 2-3, and ≥ 4).

Serum total cholesterol, HDL-cholesterol, LDL-cholesterol, and triglyceride levels were measured by an auto-analyzer (ADVIA 1800 Auto Analyzer; Siemens Medical Solutions, Malvern, PA, USA) from blood samples that were collected after at least 8 hours of fasting. All laboratory tests were performed in a central laboratory (Seoul Clinical Laboratories R&D Center, Seoul, Korea). Hypertriglyceridemia was defined as a serum triglyceride level ≥ 150 mg/dL [14]. Hypo-HDL-cholesterolemia was defined as an HDL-cholesterol level < 40 mg/dL and hyper-LDL-cholesterolemia was defined as an LDL-cholesterol level ≥ 160 mg/dL [15].

Socio-demographic information, medical history, and health-related behaviors were collected by trained interviewers following a standardized protocol in systematic face-to-face interviews. Socio-demographic factors included age, gender, education level (low: completion of secondary school or below; middle: completion of high school; high: completion of university or more), household income (divided into quartiles), and marriage (married or unmarried). Height was measured using a stadiometer (DS-102 Jenix; Seoul, Korea) to the nearest 0.1 cm, and weight was measured using a digital scale (DB-150; CAS, Seongnam, Korea) to the nearest 0.1 kg. Body mass index (BMI) was calculated as weight (kg) divided by height squared (m2). Waist circumference was measured at the middle point between the lower border of the rib cage and iliac crest. Blood pressure was recorded thrice after 5 minutes of rest in the sitting position using an automated oscillometric device placed on the right arm (HEM-7080; Omron Health, Matsusaka, Japan); the mean of 2 values (second and third) was calculated. Health-related behaviors included smoking status (never smokers, who smoked < 100 cigarettes throughout their lifetime; past smokers; and current smokers), alcohol consumption (non-drinkers, past drinkers, and current drinkers) [16], physical activity, and sleep duration. Physical activity was assessed using the Korean version of the International Physical Activity Questionnaire (IPAQ)-short form [17] where the last 7 days of activities were recorded and participants were divided into 3 groups (low, moderate, and high) following the IPAQ guidelines [18]. Sleep duration was self-reported and grouped into quartiles (< 6.8, 6.8-7.7, 7.8-8.7, and ≥ 8.8 hours). The Beck Depression Inventory II (BDI-II), which has been validated for the Korean population, was used to measure depressive symptoms [19]. The BDI-II consists of 21 items measured on a Likert scale from 0 to 3, with questions on symptoms related to depression in the previous 2 weeks. Higher scores represent more severe depressive symptoms. Nutritional information was collected using a semi-quantitative food frequency questionnaire, which was developed and validated for assessing Korean adults in the Korean National Health and Nutrition Examination Survey [20]. The intake of major macronutrients (total calories, carbohydrates, protein, and fat) was calculated.

### Statistical analysis

Since it is known that men and women have different stress responses and blood lipid distributions, all statistical analyses were performed separately for men and women. It has also been reported that the distribution of dyslipidemia among Koreans differs not only by gender, but also by menopausal status [21]. Thus, the analysis for women was further stratified into premenopausal women and postmenopausal women, as presented in Supplementary Material 4. To identify differences in general characteristics in the study population stratified by gender, the independent ttest for normally distributed continuous variables, the chi-square test for categorical variables, and the Mann–Whitney U test for continuous variables with a skewed distribution were performed. Data are presented as mean with standard deviation, median with interquartile range, or number with percentage. Multiple linear regression models were computed to examine the associations between SLEs and each category of serum lipid levels. Adjustment was performed for potential confounders, such as age, BMI, waist circumference, systolic blood pressure, diastolic blood pressure, education level, marital status, smoking, drinking, physical activity, dietary (total energy, carbohydrate, and fat) intake, and depression score. Furthermore, binary logistic regression analysis was performed along with adjustment for all potential confounders to compare serum triglyceride levels according to the number of SLEs. Statistical analyses were conducted using SAS version 9.4 (SAS Institute Inc., Cary, NC, USA), and statistical significance was set at p-value < 0.05.

### Ethics statement

This study was approved by the Institutional Review Board of Yonsei University Health System, Seoul, Korea (4-2013-0661). Before participating in the study, all participants provided written informed consent.

## RESULTS

The characteristics of all study participants (n= 2,963) divided by gender (1,076 men and 1,887 women) are presented in Table 1. The mean age of all participants was 50.2± 9.7 years. The median (interquartile range) number of total SLEs was 3 (1-5), whereas the median (interquartile range) number of SLEs with positive and negative effects was 1 (1-2) and 1 (1-3), respectively. No significant difference was found in the median number of SLEs with positive and negative effects between men and women. Hypertriglyceridemia was found in 24.8% of all participants. Men participants had a higher prevalence of hypertriglyceridemia (38.4%) than women participants (17.0%) (data not shown). The mean serum triglyceride level was 127.0 ± 86.9 mg/dL for all participants. The mean serum triglyceride level was significantly higher in men (156.0 ± 111.3 mg/dL) than in women (110.5± 63.8 mg/dL).

### Stressful life events and lipid levels

The association between different types of SLEs (including SLEs with positive effects and SLEs with negative effects) and lipid levels are presented in Table 2. We found a positive association between SLEs with negative effects (odds ratio [OR], 1.29; 95% confidence interval [CI], 1.07 to 1.72) and hypertriglyceridemia in men. However, a significant association was found for SLEs with positive effects (OR, 1.47; 95% CI, 1.14 to 1.89) in women. Furthermore, we did not find a significant association between different types of SLEs and hypo-HDL-cholesterolemia or hyper-LDL-cholesterolemia in either men or women. Table 3 presents the risk of hypertriglyceridemia according to the number of total SLEs, SLEs with positive effects, SLEs with negative effects, and their sum of scores in men and women, respectively. Men who had ≥ 4 SLEs with positive effects had a higher likelihood of having hypertriglyceridemia (OR, 2.57; 95% CI, 1.02 to 6.46) than those who had no SLEs in the past 6 months. Similar results were also observed for SLEs with negative effects after adjusting for potential confounders (OR, 1.99; 95% CI, 1.16 to 3.41). Following the same trend, an increased OR also was seen for the sum of the negative effect score (OR, 1.71; 95% CI, 1.11 to 2.55) in men. In addition, hypertriglyceridemia was associated with a per-event increase in the total number of SLEs (OR, 1.05; 95% CI, 1.01 to 1.09), the number of SLEs with negative effects (OR, 1.07; 95% CI, 1.01 to 1.13), and the sum of the negative effect score (OR, 1.04; 95% CI, 1.01 to 1.08) (Table 3). However, no significant association was observed in women (Table 3), either pre-menopausal or post-menopausal (Supplementary Material 4).

Multiple linear regression analysis of male participants showed that number of SLEs had a positive association with serum triglyceride levels. After adjusting for all confounders in a model, the total number of SLEs (β= 3.333; p= 0.001), SLEs with positive effects (β= 5.557; p= 0.007), the sum of positive affect score (β= 2.229; p = 0.041), and SLEs with negative effects (β= 3.286; p = 0.021) showed positive linear associations with serum triglyceride levels (Table 4).

## DISCUSSION

Our study results demonstrate that experiencing SLEs was associated with elevated blood triglyceride levels and the presence of hypertriglyceridemia, especially among adult men. Both positive and negative SLEs may increase blood triglyceride levels, although the association was clearer for SLEs with negative effects. However, women were more likely to show an association between SLEs with positive effects and hypertriglyceridemia. Previous studies have considered workplace stress or childhood trauma as triggers of increasing serum triglyceride levels [9,10,22]. However, in our study, we found that SLEs with either positive or negative impacts had a positive association with increased triglyceride levels. In a Finnish cohort study of 3,407 participants (1,618 men and 1,789 women), metabolic syndrome was associated with SLEs related to work or finances, and the risk was further increased with having at least 3 SLEs in any of the life-related domains assessed. However, the change in serum triglyceride levels did not show any gender stratification [23]. A Dutch cohort study of a middleaged and elderly population (n= 1,099) reported a positive association between the number of SLEs (especially financial) and the risk of developing metabolic syndrome (OR, 2.25; 95% CI, 1.17 to 4.33) [24].

The association between SLEs and hypertriglyceridemia can be explained by several possible mechanisms. One of the plausible mechanisms is a change in health behaviors after experiencing SLEs. Studies from Iran have demonstrated that a stressor in the past 6-12 months resulted in elevated triglyceride levels [7,25]. Stressors can alter health behaviors such as smoking, drinking, exercise, sleeping, and eating habits, which result in the elevation of blood triglycerides [26,27]. A cohort study conducted among 2,850 Dutch participants showed that the severity of anxiety or depression was adversely associated with smoking, which might detrimentally influence triglyceride levels [28]. Although we adjusted for lifestyle factors, we cannot exclude the effect of behavior changes.

Stressors stimulate the activity of the HPA axis, resulting in increased cortisol levels, which can elevate circulating triglyceride levels [29]. However, this response to stress is determined by the number of SLEs, the impact of an event, and the ability of an individual to adapt [30]. For example, marriage can be considered a positive event or negative event and can act as a stressor, depending on an individual’s sense of control, the stability of his or her status, or way of adaptation to life changes [31]. This offers a possible explanation for why men and women were influenced by life changes in different ways in our study. The way of adaptation to life changes might play a pivotal role in the change of serum triglyceride levels in response to SLEs with positive and negative effects in men and women. In our study, we considered the number of events instead of scrutinizing the impact of each event on serum triglyceride levels separately, as the events varied in type. We also took into account the score of each event, both positive and negative. There are no standard objective weights for SLEs; therefore, we used scores as a measure of the direction and strength of each event. We found a dose-response association between hypertriglyceridemia and an increase of SLE, especially for SLEs with negative effects and the score in men, whereas women did not show any significant association. In addition, graded associations between hypertriglyceridemia and SLEs with both positive and negative effects in men were observed, when the number of events was ≥ 4. However, we did not find a significant association between the scores of SLEs with positive effects and hypertriglyceridemia when the scores were ≥ 4. A likely explanation for this finding may be that the single event with the highest impact can affect an individual especially strongly; nevertheless, the frequency of events, regardless of the magnitude of their impact, also matters.

In our study, the relationship between SLEs and triglyceride levels was more prominent in men than in women. It has been reported that the activity of the HPA axis in response to stress is greater in men than in women [32]. Further, the metabolic clearance rate of fatty acids is higher in women than in men [33]. Although this cannot fully explain our results, we can hypothesize that physiological mechanisms may have contributed to the finding that serum triglyceride levels were higher in man participants than in woman participants in response to SLEs.

To the best of our knowledge, this study was the first to elucidate the association between SLEs and hypertriglyceridemia in a middle-aged Korean population. To minimize the possibility of distortion caused by prevalent diseases or medication use, we excluded people with major cardiovascular disease or cancer and people taking any lipid-controlling medications or other treatments that might influence serum triglyceride levels. However, the study results may also have implications for dyslipidemia patients taking lipid-lowering agents or patients with diabetes on medication for glycemic control. Prior studies have reported that stress stimulates several hormones like norepinephrine, epinephrine, cortisol, β-endorphin, and growth hormone, which can affect glucose homeostasis in healthy people and in patients with diabetes [34,35]. Moreover, hypertriglyceridemia has adverse effects on insulin sensitivity and islet beta-cell function, which might affect glycemic control [36]. Our study illustrated that SLEs can play a role in increasing serum triglyceride levels; therefore, more attention should be paid to the proper management of stressors in dyslipidemia patients taking lipid-lowering drugs and patients with diabetes. Our study measured 50 types of potential SLE experiences with their impact score, while most previous studies only included a limited number of events or evaluated only the presence or absence of SLEs.

There are several limitations of the current study. First, this is a cross-sectional analysis of baseline data of the CMERC cohort study; thus, the temporal relationship between SLEs and serum triglyceride levels could not be established. Second, data were collected by in-person interviews; measurement errors and recall bias may have affected the accuracy of the data. Third, although adjustment was conducted for potential confounders, the possibility of residual confounding remains. Fourth, we cannot rule out the possibility that the observed association of SLEs and hypertriglyceridemia in men reflects type I error due to multiple tests. Considering this limitation, we interpreted our findings as exploratory results, and further replication studies are needed for confirmation.

In conclusion, our study shows that SLEs might have adverse effects on triglyceride metabolism, especially in men. Considering the high prevalence of hypertriglyceridemia among middle-aged Korean men, adequate management of stressors in daily life might be a challenge for cardiovascular disease prevention. Further research is needed on gender differences in the association between SLEs and triglyceride levels.

## Figures and Tables

**Table 1.** General characteristics of the total study population and according to gender

Variables	Total (n=2,963)	Men (n=1,076)	Women (n=1,887)	p-value^1^
Age (yr)	50.2±9.7	49.1±10.5	50.8±9.2	<0.001
Body mass index (kg/m²)	23.8±3.1	24.8±2.9	23.1±2.9	<0.001
Waist circumference (cm)	81.3±9.2	81.6±9.5	81.1±9.1	0.112
Systolic blood pressure (mmHg)	118.5±15.1	125.4±13.8	114.5±14.4	<0.001
Diastolic blood pressure (mmHg)	76.4±10.2	81.1±10.2	73.7±9.3	<0.001
Education				
≤Middle school or less	334 (11.3)	81 (7.5)	253 (13.4)	<0.001
High school	1141 (38.5)	309 (28.7)	832 (44.1)	
≥University or more	1,488 (50.2)	686 (63.8)	802 (42.5)	
Household income (yr)				
Q1	701 (23.7)	212 (19.7)	489 (25.9)	<0.001
Q2	549 (18.5)	204 (19.0)	345 (18.3)	
Q3	926 (31.3)	347 (32.2)	579 (30.7)	
Q4	787 (26.6)	313 (29.1)	474 (25.1)	
Married	2,768 (93.4)	986 (91.6)	1,782 (94.4)	0.004
Smoking				
Never smoker	2,006 (67.7)	254 (23.6)	1,752 (92.8)	<0.001
Past smoker	523 (17.7)	448 (41.6)	75 (4.0)	
Current smoker	434 (14.6)	374 (34.8)	60 (3.2)	
Drinking				
Non-drinker	564 (19.0)	76 (7.1)	488 (25.9)	<0.001
Past drinker	135 (4.6)	63 (5.9)	72 (3.8)	
Current drinker	2,264 (76.4)	937 (87.0)	1,327 (70.3)	
Physical activity				
Low	2,209 (74.5)	828 (77.0)	1,381 (73.2)	0.001
Moderate	432 (14.6)	122 (11.3)	310 (16.4)	
High	322 (10.9)	126 (11.7)	196 (10.4)	
Sleep duration (hr)				
Q1 (<6.8)	1,273 (43.0)	436 (40.5)	837 (44.4)	0.052
Q2 (6.8-7.7)	988 (33.3)	389 (36.1)	599 (31.7)	
Q3 (7.8-8.7)	557 (18.8)	205 (19.1)	352 (18.7)	
Q4 (≥8.8)	145 (4.9)	46 (4.3)	99 (5.2)	
Energy intake (kcal/d)	2,304.2±880.2	2,737.1±984.3	2,057.4±704.6	<0.001
Carbohydrate intake (% energy)	16.3±2.1	15.7±2.4	16.7±1.9	<0.001
Protein intake (% energy)	3.3±0.6	3.1± 0.5	3.3± 0.6	<0.001
Fat intake (% energy)	1.9±0.6	1.9±0.6	2.0±0.6	<0.001
Beck Depression Index	9.6±7.1	8.2±6.4	10.5±7.4	<0.001
Lipid profile (mg/dL)				
Total cholesterol	203.6±33.8	200.4±32.8	205.3±34.2	<0.001
HDL-cholesterol	58.3±14.8	51.7±12.7	62.1±14.6	<0.001
LDL-cholesterol	119.2±31.9	117.3±32.4	120.3±31.6	0.014
Triglyceride	127.0±86.9	156.0±111.3	110.5±63.8	<0.001
Stressful life events				
Total no. of life events	3 [1-5]	3 [1-5]	2 [1-5]	0.150
Life events with positive effects	1 [1-2]	1 [1-2]	1 [1-2]	0.389
Life events with negative effects	1 [1-3]	1 [1-3]	1 [1-2]	0.430

Values are presented as mean±standard deviation, median [interquartile range], or number (%). HDL, high-density lipoprotein; LDL, low-density lipoprotein.

1 Independent t-test, chi-square test, or a non-parametric test (Mann-Whitney U test).

**Table 2.** Associations between stressful life events and lipid levels in men and women (both pre- and post-menopause)^1^

Stressful life events		n (%)	Hypertriglyceridemia	Hypo-HDL-cholesterolemia	Hyper-LDL-cholesterolemia
Men (n=1,076)					
	Total no. of life events				
	No	171 (15.9)	1.00 (reference)	1.00 (reference)	1.00 (reference)
	Yes	905 (84.1)	1.33 (0.91, 1.94)	0.74 (0.45, 1.21)	0.85 (0.48, 1.50)
	Life events with positive effects				
	No	514 (47.8)	1.00 (reference)	1.00 (reference)	1.00 (reference)
	Yes	562 (52.2)	1.16 (0.89, 1.53)	0.83 (0.59, 1.17)	0.77 (0.50, 1.17)
	Life events with negative effects				
	No	375 (34.9)	1.00 (reference)	1.00 (reference)	1.00 (reference)
	Yes	701 (65.1)	1.29 (1.07, 1.72)^*^	0.81 (0.57, 1.17)	1.23 (0.78, 1.94)
Women (n=1,887)					
	Total no. of life events				
	No	315 (16.7)	1.00 (reference)	1.00 (reference)	1.00 (reference)
	Yes	1,572 (83.3)	1.24 (0.87, 1.76)	1.33 (0.72, 2.47)	1.32 (0.87, 2.01)
	Life events with positive effects				
	No	925 (49.0)	1.00 (reference)	1.00 (reference)	1.00 (reference)
	Yes	962 (51.0)	1.47 (1.14, 1.89)^*^	0.92 (0.56, 1.51)	1.12 (0.84, 1.49)
	Life events with negative effects				
	No	674 (35.7)	1.00 (reference)	1.00 (reference)	1.00 (reference)
	Yes	1,213 (64.3)	1.15 (0.88, 1.50)	1.52 (0.92, 2.50)	1.10 (0.81, 1.49)

Values are presented as odds ratio (95% confidence interval). HDL, high-density lipoprotein; LDL, low-density lipoprotein.

1 Results were derived from binary logistic regression models; Adjusted for age, body mass index, waist circumference, systolic blood pressure, diastolic blood pressure, education, marriage, smoking, drinking, physical activity, total energy, carbohydrate intake, fat intake, and depression score.

* p<0.05.

**Table 3.** Associations between stressful life events and hypertriglyceridemia in men and women divided into subgroups^1^

Stressful life events		Total, n	With hypertriglyceridemia, n (%)	Unadjusted	p-value	Adjusted^2^	p-value
Men (n=1,076)							
	Total no. of life events (events)						
	0	171	59 (34.5)	1.00 (reference)		1.00 (reference)	
	1	162	64 (39.5)	1.24 (0.79, 1.94)	0.345	1.34 (0.82, 2.18)	0.246
	2-3	509	193 (37.9)	1.16 (0.81, 1.67)	0.424	1.28 (0.85, 1.89)	0.236
	≥4	234	97 (41.5)	1.34 (0.89, 2.02)	0.156	1.50 (0.96, 2.35)	0.075
	Per 1	1,076	413 (38.4)	1.04 (1.00, 1.08)	0.044	1.05 (1.01, 1.09)	0.023
	Life events with positive effects (events)						
	0	514	191 (37.2)	1.00 (reference)		1.00 (reference)	
	1	257	103 (40.1)	1.13 (0.83, 1.54)	0.432	1.30 (0.93, 1.83)	0.122
	2-3	281	106 (37.7)	1.02 (0.76, 1.38)	0.875	0.99 (0.71, 1.37)	0.938
	≥4	24	13 (54.2)	1.99 (0.88, 4.55)	0.099	2.57 (1.02, 6.46)	0.045
	Per 1	1,076	413 (38.4)	1.06 (0.98, 1.15)	0.134	1.07 (0.98, 1.17)	0.123
	Sum of positive effect scores						
	0	514	191 (37.2)	1.00 (reference)		1.00 (reference)	
	1	137	55 (40.1)	1.13 (0.77, 1.67)	0.522	1.44 (0.95, 2.19)	0.086
	2-3	303	118 (38.9)	1.08 (0.81, 1.45)	0.611	1.07 (0.78, 1.48)	0.672
	≥4	122	49 (40.2)	1.14 (0.76, 1.70)	0.538	1.13 (0.73, 1.75)	0.578
	Per 1 point	1,076	413 (38.4)	1.02 (0.98, 1.06)	0.401	1.02 (0.98, 1.07)	0.340
	Life events with negative effects (events)						
	0	375	133 (35.5)	1.00 (reference)		1.00 (reference)	
	1	240	100 (41.7)	1.30 (0.93, 1.81)	0.122	1.43 (0.99, 2.05)	0.052
	2-3	384	141 (36.7)	1.06 (0.79, 1.42)	0.725	1.13 (0.82, 1.56)	0.453
	≥4	77	39 (50.6)	1.87 (1.14, 3.06)	0.013	1.99 (1.16, 3.41)	0.012
	Per 1	1,076	413 (38.4)	1.06 (0.99, 1.11)	0.054	1.07 (1.01, 1.13)	0.034
	Sum of negative effect scores						
	0	375	133 (35.5)	1.00 (reference)		1.00 (reference)	
	1	172	66 (38.4)	1.13 (0.78, 1.65)	0.512	1.16 (0.77, 1.74)	0.476
	2-3	362	136 (37.6)	1.09 (0.81, 1.48)	0.553	1.22 (0.88, 1.69)	0.234
	≥4	167	78 (46.7)	1.60 (1.10, 2.31)	0.014	1.71 (1.11, 2.55)	0.008
	Per 1 point	1,076	413 (38.4)	1.03 (0.99, 1.06)	0.071	1.04 (1.01, 1.08)	0.028
Women (n=1,887)							
	Total no. of life events (events)						
	0	315	46 (14.6)	1.00 (reference)		1.00 (reference)	
	1	334	55 (16.5)	1.15 (0.75, 1.77)	0.513	1.09 (0.70, 1.70)	0.706
	2-3	864	151 (17.5)	1.24 (0.87, 1.77)	0.242	1.25 (0.86, 1.82)	0.238
	≥4	374	69 (18.4)	1.32 (0.88, 1.97)	0.178	1.41 (0.92, 2.16)	0.116
	Per 1	1,887	321 (17.0)	1.01 (0.97, 1.05)	0.812	1.02 (0.97, 1.06)	0.506
	Life events with positive effects (events)						
	0	925	138 (14.9)	1.00 (reference)		1.00 (reference)	
	1	451	91 (20.2)	1.44 (1.08, 1.93)	0.014	1.55 (1.14, 2.10)	0.005
	2-3	475	87 (18.3)	1.28 (0.95, 1.72)	0.102	1.41 (1.04, 1.93)	0.029
	≥4	36	5 (13.9)	0.92 (0.35, 2.41)	0.865	1.01 (0.36, 2.73)	0.976
	Per 1	1,887	321 (17.0)	1.02 (0.94, 1.10)	0.748	1.03 (0.95, 1.12)	0.431
	Sum of positive effect score						
	0	925	138 (14.9)	1.00 (reference)		1.00 (reference)	
	1	231	52 (22.5)	1.66 (1.16, 2.37)	0.006	1.70 (1.17, 2.47)	0.006
	2-3	515	95 (18.4)	1.29 (0.97, 1.72)	0.082	1.47 (1.09, 1.99)	0.013
	≥4	216	36 (16.7)	1.14 (0.76, 1.70)	0.520	1.22 (0.80, 1.85)	0.363
	Per 1 point	1,887	321 (17.0)	1.08 (0.97, 1.20)	0.162	1.12 (1.01, 1.26)	0.044
	Life events with negative effects (events)						
	0	674	106 (15.7)	1.00 (reference)		1.00 (reference)	
	1	421	79 (18.8)	1.24 (0.90, 1.71)	0.192	1.15 (0.82, 1.60)	0.427
	2-3	689	121 (18.6)	1.14 (0.86, 1.52)	0.364	1.20 (0.89, 1.62)	0.233
	≥4	103	15 (14.6)	0.91 (0.51, 1.64)	0.762	0.92 (0.52, 1.89)	0.777
	Per 1	1,887	321 (17.0)	1.01 (0.99, 1.06)	0.877	1.01 (0.94, 1.07)	0.916
	Sum of negative effect score						
	0	674	106 (15.7)	1.00 (reference)		1.00 (reference)	
	1	304	62 (20.4)	1.37 (0.97, 1.94)	0.074	1.23 (0.85, 1.77)	0.269
	2-3	620	102 (16.5)	1.06 (0.78, 1.42)	0.723	1.13 (0.82, 1.54)	0.459
	≥4	289	51 (17.6)	1.15 (0.80, 1.66)	0.460	1.10 (0.75, 1.62)	0.619
	Per 1 point	1,887	321 (17.0)	1.03 (0.92, 1.15)	0.623	1.04 (0.92, 1.16)	0.550

Values are presented as odds ratio (95% confidence interval).

1 Results were derived from binary logistic regression models.

2 Adjusted for age, body mass index, waist circumference, systolic blood pressure, diastolic blood pressure, education, marriage, smoking, drinking, physical activity, total energy, carbohydrate intake, fat intake, and depression score.

**Table 4.** Association between stressful life events and triglyceride levels in men and women^1^

Stressful life events	Men	p-value	Women	p-value
Total no. of life events	3.333 (1.381, 5.285)	0.001	0.451 (-0.471, 1.373)	0.338
Life events with positive effects	5.557 (1.499, 9.616)	0.007	0.766 (-1.059, 2.592)	0.410
Sum of positive effect score	2.229 (0.103, 4.456)	0.041	0.452 (-0.506, 1.410)	0.355
Life events with negative effects	3.286 (0.491, 6.081)	0.021	0.126 (-1.203, 1.456)	0.852
Sum of negative effect score	1.388 (-0.275, 3.015)	0.093	0.053 (-0.726, 0.832)	0.133

Values are presented as β (95% confidence interval).

1 Results were derived from multiple linear regression models; Adjusted for age, body mass index, waist circumference, systolic blood pressure, diastolic blood pressure, education, marriage, smoking, drinking, physical activity, total energy, carbohydrate intake, fat intake, and depression score.

## SUPPLEMENTARY MATERIALS

Supplementary Material 1. General characteristics of participants in the baseline examination of CMERC cohort and comparison in both center

Supplementary Material 2. Flowchart of selecting process of participants for this study.

Supplementary Material 3. Distribution of 50 types of stressful life events

Supplementary Material 4. Association between stressful life events and hypertriglyceridemia in premenopausal and postmenopausal women.
