# Supplementary material for: Stressful life events and serum triglyceride levels: the Cardiovascular and Metabolic Diseases Etiology Research Center cohort in Korea
Source: Epidemiol Health. 2021 Jun 9;43:e2021042. doi: 10.4178/epih.e2021042 (PMC8289470; doi:10.4178/epih.e2021042)
Supplement: Supplementary Material 1. — General characteristics of participants in the baseline examination of CMERC cohort and comparison in both center [file epih-43-e2021042-suppl.docx]

**Supplementary Material 1. General characteristics of participants in the baseline examination of CMERC cohort and comparison in both center**

| **Variables** | **Center 1** | **Center 2** | **p-value*** |
| --- | --- | --- | --- |
|  | **(n= 4060)** | **(n= 4037)** |  |
| Age, year | 51.3 ± 9.4 | 51.5 ± 8.0 | 0.254 |
| Body mass index, kg/m² | 23.9 ± 3.1 | 24.3 ± 3.0 | <0.001 |
| Waist circumference, cm | 81.1 ± 9.1 | 83.5 ± 8.9 | <0.001 |
| Systolic blood pressure, mmHg | 118.6 ± 14.9 | 119.9 ± 15.0 | <0.001 |
| Diastolic blood pressure, mmHg | 76.2 ± 9.9 | 76.0 ± 10.1 | 0.569 |
| Education |  |  |  |
| Middle school or less | 580 (14.3) | 617 (15.3) | <0.001 |
| High school | 1585 (39.0) | 1918 (47.5) |  |
| University or more | 1894 (46.7) | 1502 (37.2) |  |
| Household income (year) |  |  |  |
| Q1 | 1009 (25.0) | 1117 (27.7) | 0.097 |
| Q2 | 1342 (33.3) | 1206 (29.9) |  |
| Q3 | 737 (18.3) | 741 (18.4) |  |
| Q4 | 944 (23.4) | 965 (24.0) |  |
| Married | 3841 (94.4) | 3940 (97.6) | <0.001 |
| Smoking |  |  |  |
| Never smoker | 2780 (68.5) | 2822 (69.9) | 0.232 |
| Past smoker | 724 (17.8) | 711 (17.6) |  |
| Current smoker | 556 (13.7) | 504 (12.5) |  |
| Drinking |  |  |  |
| Non-drinker | 906 (22.3) | 1137 (28.2) | <0.001 |
| Past drinker | 184 (4.5) | 118 (2.9) |  |
| Current drinker | 2970 (73.2) | 2782 (68.9) |  |
| Physical Activity |  |  |  |
| Low | 3036 (74.8) | 3039 (75.3) | 0.066 |
| Moderate | 576 (14.2) | 611 (15.1) |  |
| High | 448 (11.0) | 387 (9.6) |  |
| Sleep duration |  |  |  |
| Q1 (<6.8h) | 1729 (42.6) | 1384 (34.3) | 0.187 |
| Q2 (6.8-7.7h) | 1345 (33.1) | 1423 (35.2) |  |
| Q3 (7.8-8.7h) | 770 (19.0) | 939 (23.3) |  |
| Q4 (≥ 8.8 h) | 216 (5.3) | 291 (7.2) |  |
| Energy intake, kcal/day | 2278.3 ± 870.8 | 2287.9 ± 746.0 | 0.613 |
| Carbohydrate intake (% energy) | 16.4 ± 2.1 | 16.9 ± 2.0 | <0.001 |
| Protein intake (% energy) | 3.3 ± 0.6 | 3.2 ± 0.6 | <0.001 |
| Fat intake (% energy) | 1.9 ± 0.6 | 1.8 ± 0.6 | <0.001 |
| Beck depression index (BDI) | 9.9 ± 7.3 | 11.1 ± 7.5 | <0.001 |
| Lipid profile, mg/dL |  |  |  |
| Total cholesterol | 198.4 ± 35.4 | 191.8 ± 33.7 | <0.001 |
| HDL cholesterol | 58.2 ± 14.7 | 54.2 ± 13.2 | <0.001 |
| LDL cholesterol | 115.3 ± 32.1 | 110.6 ± 31.2 | <0.001 |
| Triglyceride | 127.9 ± 87.8 | 135.4 ± 97.7 | <0.001 |
| Stressful life events |  |  |  |
| Total number of life events | 3 [ 1-5 ] | 3 [ 1-5 ] | 0.153 |
| Positively affecting life events | 1 [ 1-2 ] | 1 [ 0-2 ] | <0.001 |
| Negatively affecting life events | 2 [ 1-4 ] | 2 [ 1-4 ] | 0.233 |

Values are presented as mean ± SD, median [interquartile range], or number (%).

HDL-cholesterol, high-density lipoprotein cholesterol; LDL-cholesterol, low-density lipoprotein cholesterol.

^a^ P value are calculated by independent t-tests, chi-square tests or non-parametric test (Mann-Whitney u test).

CMERC center 1

Initial population

N=4060

Excluding people with missing information for

Stressful Life Event (n=1)

Triglyceride (n=2)

Blood Pressure (n =2)

Waist circumference (n=2)

Income (n=28)

Total energy intake, carbohydrate, protein & fat intake (n=460)

Excluding people with

Lipid lowering drugs (n=461)

Diabetes (n=216)

Kidney disease (n=7)

Valid information for key variables

N=3565

Final Inclusion N=2963

(1076 men, 1088 pre-menopausal women, 799 post-menopausal women)

**Supplementary Material 2. Flowchart of selecting process of participants for this study.**

**Supplementary Material 3. Distribution of 50 types of stressful life events**

| **No.** | **Stressful life events** | | **Total** | **Affect in a positive way** | **No impact** | **Affect in a negative way** |
| --- | --- | --- | --- | --- | --- | --- |
|  |  |  | **N (%)** | **N (%)** | **N (%)** | **N (%)** |
| **1** | Marriage (formal commitment in a relationship) | | 21 (0.7) | 19 (0.6) | 2 (0.1) | 0 |
| **2** | Detention in a jail or comparable institution | | 0 | 0 | 0 | 0 |
| **3** | Death of spouse | | 5 (0.1) | 0 | 0 | 5 (0.1) |
| **4** | Major change in sleeping habit ( much more or less sleep ) | | 790 (26.7) | 84 (3.1) | 107 (3.6) | 599 (20.2) |
| **5** | Death of a close family member | |  |  |  |  |
|  |  | Father | 34 (1.1) | 1 (0.03) | 4 (0.1) | 29 (0.97) |
|  |  | Mother | 25 (0.8) | 0 | 3 (0.1) | 22 (0.7) |
|  |  | Brother | 20 (0.7) | 0 | 2 (0.1) | 18 (0.6) |
|  |  | Sister | 29 (1.0) | 3 (0.1) | 8 (0.3) | 18 (0.6) |
|  |  | Grandmother | 4 (0.1) | 0 | 1 (0.03) | 3 (0.1) |
|  |  | Grandfather | 137 (4.6) | 3 (0.1) | 30 (1.0) | 104 (3.5) |
|  |  | Other (specify) |  |  |  |  |
| **6** | Major change in eating habit ( much more or less sleep ) | | 685 (23.1) | 226 (7.6) | 99 (3.3) | 360 (12.1) |
| **7** | Foreclosure on mortgage or loan | | 49 (1.7) | 0 | 5 (0.2) | 44 (1.5) |
| **8** | Death of a close friend | | 97 (3.3) | 1 (0.1) | 11 (0.4) | 85 (2.8) |
| **9** | Outstanding personal achievement | | 693 (23.4) | 646 (21.8) | 17 (0.6) | 30 (1.0) |
| **10** | Minor law violation (traffic tickets, disturbing the peace etc) | | 295 (10.0) | 5 (0.2) | 76 (2.6) | 214 (7.2) |
| **11** | Male: Wife/girlfriend's pregnancy | | 17 (0.6) | 13 (0.4) | 1 (0.1) | 3 (0.1) |
| **12** | Female: Pregnancy | | 8 (0.3) | 5 (0.2) | 0 | 3 (0.1) |
| **13** | Changed work situation | | 676 (22.8) | 279 (9.4) | 80 (2.7) | 317 (10.7) |
|  |  | (different work responsibility, major change in working conditions, working hours, etc) |  |  |  |  |
| **14** | New job | | 212 (7.2) | 146 (4.9) | 22 (0.7) | 44 (1.5) |
| **15** | Serious illness of a close family member | |  |  |  |  |
|  |  | Father | 192 (6.5) | 2 (0.1) | 12 (0.4) | 178 (6.0) |
|  |  | Mother | 103 (3.5) | 0 | 9 (0.3) | 94 (3.2) |
|  |  | Brother | 81 (2.7) | 0 | 5 (0.2) | 76 (2.5) |
|  |  | Sister | 2 (0.1) | 0 | 0 | 2 (0.1) |
|  |  | Grandmother | 10 (0.3) | 1 (0.1) | 1 (0.1) | 8 (0.2) |
|  |  | Grandfather | 85 (2.9) | 0 | 4 (0.1) | 81 (2.8) |
|  |  | Other (specify) | 75 (2.5) | 0 | 3 (0.1) | 72 (2.4) |
| **16** | Sexual difficulties | | 207 (7.0) | 7 (0.2) | 16 (0.5) | 189 (6.2) |
| **17** | Trouble with employer | | 202 (6.8) | 9 (0.3) | 13 (0.4) | 180 (6.1) |
|  |  | (In danger of losing job, being suspended, demoted, etc.) |  |  |  |  |
| **18** | Problems with in-laws | | 254 (8.6) | 8 (0.3) | 17 (0.6) | 229 (7.7) |
| **19** | Major change in financial status (a lot better off or a lot worse off) | | 419 (14.1) | 66 (2.2) | 16 (0.5) | 337 (11.4) |
| **20** | Major change in closeness of family members (increased or decreased closeness) | | 342 (11.5) | 131 (4.4) | 18 (0.6) | 193 (6.5) |
| **21** | Gaining a new family member | | 189 (6.4) | 176 (5.9) | 5 (0.2) | 8 (0.2) |
|  |  | (through birth, adoption, family member moving in, etc) |  |  |  |  |
| **22** | Change of residence | | 211 (7.1) | 150 (5.1) | 34 (1.1) | 27 (0.9) |
| **23** | Marital separation from mate (due to conflicts) | | 19 (0.6) | 3 (0.1) | 0 | 16 (0.5) |
| **24** | Major change in church activities (increased or decreased attendance) | | 193 (6.5) | 121 (4.1) | 21 (0.7) | 51 (1.7) |
| **25** | Marital reconciliation with mate | | 152 (5.1) | 98 (3.3) | 16 (0.5) | 38 (1.3) |
| **26** | Major change in number of arguments with spouse | | 675 (22.8) | 126 (4.3) | 119 (4.0) | 430 (14.5) |
|  |  | (a lot more or a lot less arguments) |  |  |  |  |
| **27** | Married male: Change in wife's work outside of home | | 68 (2.3) | 29 (0.9) | 21 (0.7) | 18 (0.6) |
|  |  | (beginning work, ceasing work, changing new job, etc) |  |  |  |  |
| **28** | Married female: Changes in husband's work | | 132 (4.5) | 41 (1.4) | 30 (1.0) | 61 (2.1) |
|  |  | (loss of job, beginning new job, retirement, etc) |  |  |  |  |
| **29** | Major change in usual type and/or amount of recreation | | 353 (11.9) | 255 (8.6) | 28 (0.9) | 70 (2.4) |
| **30** | Borrowing more than 1000000 (buying home, business etc) | | 272 (9.2) | 29 (0.9) | 84 (2.8) | 159 (5.4) |
| **31** | Borrowing less than 1000000 won (buying home, business etc) | | 166 (5.6) | 6 (0.2) | 54 (1.8) | 106 (3.6) |
| **32** | Being fired from job | | 23 (0.8) | 1 (0.1) | 1 (0.1) | 21 (0.6) |
| **33** | Male: Wife/girlfriend's abortion | | 8 (0.3) | 0 | 0 | 8 (0.3) |
| **34** | Female: Having abortion | | 6 (0.2) | 0 | 0 | 6 (0.2) |
| **35** | Major personal illness or injury | | 137 (4.6) | 4 (0.2) | 9 (0.3) | 124 (4.2) |
| **36** | Major change in social activities e.g ( parties, movies, visiting ) | | 512 (17.3) | 303 (16.2) | 81 (2.7) | 128 (4.3) |
|  |  | (increased or decreased participation) |  |  |  |  |
| **37** | Major change in living conditions of family | | 219 (7.4) | 167 (5.6) | 29 (1.0) | 23 (0.7) |
|  |  | (building new home, remodelling, deterioration of home, neighbourhood etc) |  |  |  |  |
| **38** | Divorce | | 8 (0.3) | 2 (0.1) | 1 (0.01) | 5 (0.2) |
| **39** | Serious injury or illness of close friend | | 114 (3.8) | 2 (0.1) | 3 (0.1) | 109 (3.6) |
| **40** | Retirement from work | | 94 (3.2) | 25 (0.8) | 22 (0.7) | 47 (1.6) |
| **41** | Son or daughter leaving home (due to marriage, college etc) | | 239 (8.1) | 92 (3.1) | 73 (2.5) | 74 (2.5) |
| **42** | Ending of formal schooling | | 26 (0.9) | 17 (0.6) | 6 (0.2) | 3 (0.1) |
| **43** | Separation from spouse (due to work, travel etc) | | 149 (5.0) | 35 (1.2) | 67 (2.3) | 47 (1.6) |
| **44** | Engagement | | 7 (0.2) | 0 | 0 | 7 (0.2) |
| **45** | Breaking up with boyfriend/girlfriend | | 28 (0.9) | 0 | 4 (0.1) | 24 (0.8) |
| **46** | Leaving home for the first time | | 20 (0.7) | 11 (0.4) | 3 (0.1) | 6 (0.2) |
| **47** | Reconciliation with boyfriend/girlfriend | | 22 (0.7) | 12 (0.4) | 7 (0.2) | 3 (0.1) |
| **48** | Other recent experiences which have had an impact on your life (list and rate 1) | | 122 (4.1) | 31 (1.1) | 5 (0.2) | 85 (2.8) |
| **49** | Other recent experiences which have had an impact on your life (list and rate 2) | | 23 (0.8) | 18 (0.7) | 1 (0.01) | 4 (0.1) |
| **50** | Other recent experiences which have had an impact on your life (list and rate 3) | | 5 (0.2) | 2 (0.1) | 0 | 3 (0.1) |

Values are presented as number (%).

**Supplementary Material 4. Association between stressful life events and hypertriglyceridemia in premenopausal and postmenopausal women**.

| **Stressful life events** | | **Total, n** | **With hypertriglyceridemia**  **n (%)** |  | **Unadjusted** | | |  | **Adjusted ^2^** | | |
| --- | --- | --- | --- | --- | --- | --- | --- | --- | --- | --- | --- |
|  |  |  |  |  | **OR** | **( 95% CI )** | ***p-*value** |  | **OR** | **( 95% CI )** | ***p-*value** |
| **Premenopausal women** | |  |  |  |  |  |  |  |  |  |  |
| Total no. of life events (events) | |  |  |  |  |  |  |  |  |  |  |
|  | 0 | 168 | 24 (14.3) |  |  | Reference |  |  |  | Reference |  |
|  | 1 | 170 | 29 (17.1) |  | 1.23 | ( 0.69 - 2.22 ) | 0.484 |  | 1.18 | ( 0.64 - 2.19 ) | 0.598 |
|  | 2-3 | 496 | 94 (19.0) |  | 1.40 | ( 0.86 - 2.28 ) | 0.173 |  | 1.38 | ( 0.83 - 2.29 ) | 0.216 |
|  | ≥ 4 | 254 | 44 (17.3) |  | 1.26 | ( 0.73 - 2.16 ) | 0.407 |  | 1.34 | ( 0.76 - 2.37 ) | 0.312 |
|  | Per 1 | 1088 | 191 (17.6) |  | 1.00 | ( 0.95 - 1.05 ) | 0.997 |  | 0.99 | ( 0.94 - 1.04 ) | 0.706 |
| Life events with positive effects | |  |  |  |  |  |  |  |  |  |  |
|  | 0 | 497 | 75 (15.1) |  |  | Reference |  |  |  | Reference |  |
|  | 1 | 262 | 60 (22.9) |  | 1.67 | ( 1.14 - 2.44 ) | 0.008 |  | 1.79 | ( 1.20 - 2.68 ) | 0.004 |
|  | 2-3 | 302 | 52 (17.2) |  | 1.17 | ( 0.79 - 1.72 ) | 0.425 |  | 1.34 | ( 0.89 - 2.01 ) | 0.166 |
|  | ≥ 4 | 27 | 4 (14.8) |  | 0.98 | ( 0.33 - 2.91 ) | 0.969 |  | 1.04 | ( 0.33 - 3.27 ) | 0.945 |
|  | Per 1 | 1088 | 191 (17.6) |  | 1.02 | ( 0.92 - 1.13 ) | 0.752 |  | 0.99 | ( 0.90 - 1.10 ) | 0.920 |
| Life events with negative effects | |  |  |  |  |  |  |  |  |  |  |
|  | 0 | 373 | 59 (15.8) |  |  | Reference |  |  |  | Reference |  |
|  | 1 | 229 | 45 (19.7) |  | 1.30 | ( 0.85 - 1.99 ) | 0.228 |  | 1.19 | ( 0.75 - 1.87 ) | 0.461 |
|  | 2-3 | 409 | 79 (19.3) |  | 1.27 | ( 0.88 - 1.85 ) | 0.201 |  | 1.33 | ( 0.89 - 1.97 ) | 0.154 |
|  | ≥ 4 | 77 | 8 (10.4) |  | 0.62 | ( 0.28 - 1.35 ) | 0.227 |  | 0.61 | ( 0.27 -1.38 ) | 0.237 |
|  | Per 1 | 1088 | 191 (17.6) |  | 0.99 | ( 0.91 - 1.06 ) | 0.710 |  | 0.98 | ( 0.91 - 1.05 ) | 0.491 |
| **Postmenopausal women** | |  |  |  |  |  |  |  |  |  |  |
| Total no. of life events (events) | |  |  |  |  |  |  |  |  |  |  |
|  | 0 | 147 | 22 (15.0) |  |  | Reference |  |  |  | Reference |  |
|  | 1 | 164 | 26 (15.9) |  | 1.07 | ( 0.58 - 1.98 ) | 0.829 |  | 0.94 | ( 0.48 - 1.84 ) | 0.857 |
|  | 2-3 | 368 | 57 (15.5) |  | 1.04 | ( 0.61- 1.78 ) | 0.882 |  | 1.06 | ( 0.59 - 1.88 ) | 0.851 |
|  | ≥ 4 | 120 | 25 (20.8) |  | 1.49 | ( 0.79 - 2.81 ) | 0.212 |  | 1.55 | ( 0.79 - 3.08 ) | 0.206 |
|  | Per 1 | 799 | 130 (16.3) |  | 1.03 | ( 0.96 - 1.11 ) | 0.417 |  | 1.04 | ( 0.96 - 1.12 ) | 0.322 |
| Life events with positive effects | |  |  |  |  |  |  |  |  |  |  |
|  | 0 | 428 | 63 (14.7) |  |  | Reference |  |  |  | Reference |  |
|  | 1 | 189 | 31 (16.4) |  | 1.14 | ( 0.71 - 1.82 ) | 0.592 |  | 1.18 | ( 0.71 - 1.97 ) | 0.513 |
|  | 2-3 | 173 | 35 (20.2) |  | 1.47 | ( 0.93 - 2.32 ) | 0.099 |  | 1.58 | ( 0.96 - 2.59 ) | 0.072 |
|  | ≥ 4 | 9 | 1 (11.1) |  | 0.72 | ( 0.09 - 5.89 ) | 0.763 |  | 0.70 | ( 0.08 - 6.13 ) | 0.749 |
|  | Per 1 | 799 | 130 (16.3) |  | 1.04 | ( 0.91 - 1.12 ) | 0.558 |  | 1.05 | ( 0.91 - 1.22 ) | 0.478 |
| Life events with negative effects | |  |  |  |  |  |  |  |  |  |  |
|  | 0 | 301 | 47 (15.6) |  |  | Reference |  |  |  | Reference |  |
|  | 1 | 192 | 34 (17.7) |  | 1.16 | ( 0.72 - 1.89 ) | 0.541 |  | 1.06 | ( 0.63 - 1.79 ) | 0.815 |
|  | 2-3 | 280 | 42 (15.0) |  | 0.95 | ( 0.61 - 1.49 ) | 0.837 |  | 1.02 | ( 0.63 - 1.66 ) | 0.928 |
|  | ≥ 4 | 26 | 7 (26.9) |  | 1.99 | ( 0.79 - 5.00) | 0.143 |  | 1.99 | ( 0.72 - 5.53 ) | 0.187 |
|  | Per 1 | 799 | 130 (16.3) |  | 1.03 | ( 0.93 - 1.15 ) | 0.525 |  | 1.04 | ( 0.93 - 1.16 ) | 0.482 |

Values are presented as odds ratio (95% confidence interval).

^1^ Results were derived from binary logistic regression models

^2^ Adjusted for age, body mass index, waist circumference, systolic blood pressure, diastolic blood pressure, education, marriage, smoking, drinking, physical activity, total energy, carbohydrate intake, fat intake, and depression score.
